# Spatial Heterogeneity and Attribution Analysis of Urban Thermal Comfort in China from 2000 to 2020

**DOI:** 10.3390/ijerph19095683

**Published:** 2022-05-07

**Authors:** Jiansheng Wu, Xuechen Li, Si Li, Chang Liu, Tengyun Yi, Yuhao Zhao

**Affiliations:** 1 Key Laboratory for Urban Habitat Environmental Science and Technology, School of Urban Planning and Design, Peking University, Shenzhen 518055, China; 2001212695@stu.pku.edu.cn (X.L.); 1801213212@pku.edu.cn (S.L.); clara-liu@pku.edu.cn (C.L.); tengyun_yi@stu.pku.edu.cn (T.Y.); zhaoyh2017@pku.edu.cn (Y.Z.); 2 Laboratory for Earth Surface Processes, Ministry of Education, College of Urban and Environmental Sciences, Peking University, Beijing 100871, China

**Keywords:** thermal environment, urban thermal comfort, spatial heterogeneity, geographic detector, China

## Abstract

Research on urban thermal environments based on thermal comfort can help formulate effective measures to improve urban thermal and human settlement environments, which is of great significance for improving urban quality, urban climate change adaptation, and sustainable development. Taking 344 municipal administrative districts in China as study areas, the Universal Thermal Climate Index (UTCI) of each city in the last 20 years was calculated to evaluate thermal comfort. We then analyzed the thermal comfort and spatiotemporal heterogeneity of each city during a typical heat wave. Finally, the driving forces of the potential socioeconomic, natural, and landscape factors influencing thermal comfort were analyzed using geographic detectors. The results show that the thermal comfort index had similar spatial patterns and differentiation characteristics in different years, and the interannual variation was not obvious. Cities in the typical heat wave period were mainly distributed in East and Northwest China. The driving factor in the contribution rate of the same index in different years was basically the same and was not affected by the change in years, and the highest contribution rate was the natural factor.

## 1. Introduction

Changes in the urban thermal environment not only affect urban air quality, energy consumption, and ecosystem process evolution, but also seriously affect the health status of residents [1,2]. Especially in the scenario of global warming, extreme high-temperature events occur frequently in cities, and the morbidity and mortality of residents increase [3,4,5,6]. Changes in the thermal environment can directly or indirectly affect the health status of residents. However, increasing the frequency and intensity of extreme weather events, such as high temperatures, leads to increased exposure to high temperatures, which directly affects human health [7,8]. According to research projections, the rise in global warming from 1.5 °C to 2 °C will lead to the death of more than 279,000 urban residents due to high temperatures every year in China [9]. However, an increase in adverse meteorological conditions leads to the deterioration of air quality, which indirectly affects human health [10]. For example, under the forecast scenario of typical concentration path 4.5 (RCP4.5), by the middle of the century, the global temperature rise will increase the per capita exposure concentration of PM2.5 and ozone by 3–4% in China, and the number of premature deaths resulting from this increase is expected to be approximately 20,000 per year [11]. Therefore, in the process of continuous urbanization, clarifying the change law of the urban thermal environment and formulating corresponding measures to improve the quality of the urban thermal environment is of important theoretical and practical significance.

One important way to evaluate the urban thermal environment is through people’s subjective feelings. Compared with direct evaluation of the thermal environment by air temperature or surface temperature, the thermal comfort index can better reflect the quality of urban open spaces and outdoor attraction to people [12,13]. The factors affecting thermal comfort mainly include urban characteristics, climate, and the subjective human thermal state. Urban characteristic elements can be further divided into urban geometric, landscape, natural, social, and economic elements [14,15]. In addition, the influence of climate on outdoor thermal comfort is evident. For example, air temperature has been proved to be the most important climate factor [16,17,18]. In the subjective thermal state of human beings, different lifestyles, behaviors, cultures, tolerance, and adaptability may lead to different views of thermal comfort [14,19]. These studies have provided objective and reliable assessments of urban thermal environments. However, the time and space scales need to be further expanded, and more detailed studies are required to obtain more comprehensive results. In addition, multi-factor analysis and dominant factor identification are seldom used in the analysis of the factors influencing thermal comfort.

Previous studies on thermal comfort can be roughly summarized in the following three ways: (1) The applicability of the outdoor thermal comfort index can be explored and the index modified [20,21,22]. The main purpose of this type of research is to provide scientific guidance for the selection and application of indicators and realize the localization application of indicators. (2) Spatial distribution and factors influencing thermal comfort are analyzed. For example, based on thermal comfort evaluation results, the influencing factors of its temporal and spatial variability are further analyzed to formulate strategies for improving outdoor thermal comfort and the thermal environment [14,15,23]. At present, climate, human activities, and urban characteristics are generally considered to be important factors affecting outdoor thermal comfort [24,25,26]. (3) Exploring the impact of thermal comfort on human activities or urban outdoor space utilization is also an important research direction [27,28,29].

Studies on outdoor thermal comfort mostly establish outdoor thermal comfort indicators to represent the correlation between the outdoor environment and human thermal sensation, and to evaluate and predict human thermal sensation in a specific outdoor climate [29,30,31,32]. In 2000, the Universal Thermal Climate Index (UTCI) was proposed. This index adopts a multi-node model and considers a variety of meteorological variables (such as air temperature, mean radiation temperature, wind speed, humidity, etc.), in addition to environmental factors, human physiological characteristics, heat resistance of clothing, and other factors and variables [33,34]. The UTCI also introduces a sensitivity analysis of changes in meteorological variables, enhancing its applicability for evaluating thermal comfort under different climatic backgrounds [35].

Based on the UTCI thermal comfort index, historical monitoring data of meteorological stations with 3 h resolution and solar radiation data were used to evaluate urban thermal comfort during the years 2000, 2005, 2010, 2015, and 2019 with 344 municipal administrative regions in China as research areas, and to differentiate the spatiotemporal variation rules of urban thermal comfort in China. On this basis, the leading and important driving factors of urban thermal comfort were identified. It is helpful to seek effective measures to improve urban outdoor thermal comfort and enhance adaptation to urban climate change.

## 2. Materials and Methods

### 2.1. Study Area

**Figure 1:**
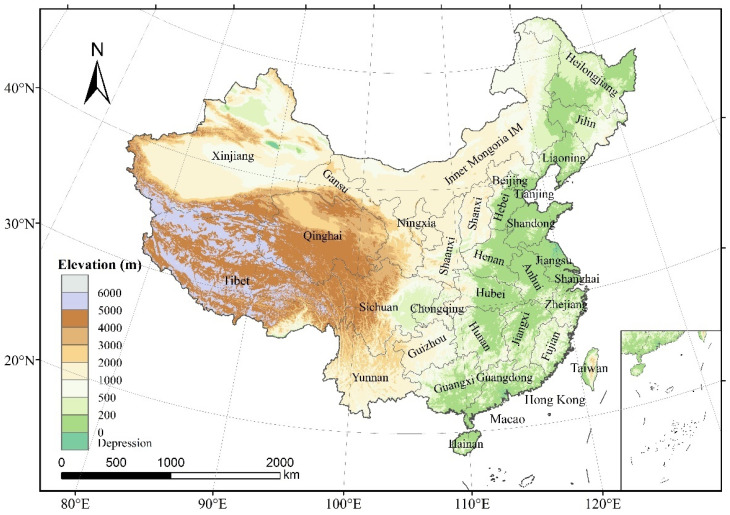
Geographical locations and topography of China.

China is located in the southeast of Eurasia, with geographic coordinates ranging from 30°52′ to 53°31′ N latitude and 73°40′ to 135°05′ E longitude (Figure 1). The land area of China is about 9.6 million km^2^, and the water area of the inland and border seas are approximately 4.7 million km^2^. The land and sea boundary reaches 40,000 km, with a vast territory, complex landforms, and diverse climates. Natural resources are abundantly available. Urbanization has been accelerating since the reform and opening up, leading to significant changes in China’s ecological environment [36,37].

### 2.2. Available Data and Technical Route

**Figure 2:**
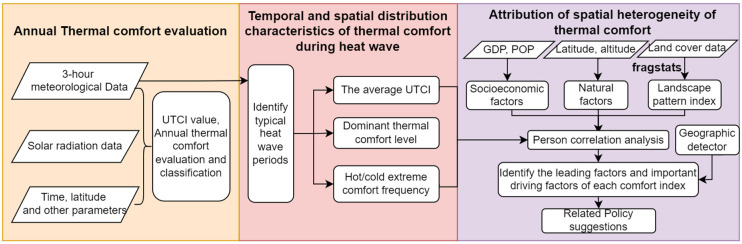
Technical roadmap of the paper.

The data sources and technical roadmap of this study are shown in Figure 2.

(1) Calculating UTCI values requires meteorological and solar radiation data. The data of temperature, dew point temperature, and wind speed from March to December in 2000, 2005, 2010, 2015, and 2019 and from January to February in 2001, 2006, 2011, 2016, and 2020 were selected as well as the solar radiation data.

Three hours of meteorological data were collected from the National Climatic Data Center (NCDC); the solar radiation data from the Climate Data website (https://cds.climate.copernicus.eu, accessed on 1 September 2020), at a temporal resolution of one hour and a spatial resolution of 0.1° × 0.1°, are shown.

(2) Other data: The land use and administrative data are from the Chinese Geographical Conditions Monitoring cloud platform (http://www.dsac.cn/DataProduct/Index/200827, accessed on 1 September 2020). Social and economic data were selected from representative GDP and population density (POP), with numerical units of CNY 10,000/km^2^ and person/km^2^, respectively. Natural factor data were obtained from the Resources and Environmental Science and Data Center of the Chinese Academy of Sciences (https://www.resdc.cn/ accessed on 1 September 2020).

### 2.3. Universal Thermal Climate Index (UTCI)

In this study, UTCI, a universal thermal climate index, was selected to evaluate the thermal comfort of cities in China. The UTCI refers to the physiological equivalent temperature calculated by combining the multi-node thermal regulation model with the adaptive clothing model, which is realized by a model based on the six-degree polynomial regression function [38]. The calculation formula is shown in Equation (1).

(1) $$UTCI=f\left(Ta;Tmrt;v_{10};RH\right)=Ta+offset\left(Ta;Tmrt;\;v_{10};RH\right)$$

where Ta (°C) is the temperature, RH (%) is the relative humidity, Tmrt (°C) is the average radiation temperature, and $v_{10}$ (m/s) is the wind speed measured at 10 m height. The value of Tmrt cannot be obtained directly and needs to be calculated using Formula (2).

(2) $$Tmrt=\frac{{\left(R_{prim}+0.5Lg+0.5La\right)}^{\frac{1}{4}}}{\left(0.95\times 5.667\times 10^{-8}\right)}-273$$

(3) $$La=5.5\times 10^{-8}\times {\left(273+Ta\right)}^{4}\times [0.82-0.25\times 10^{\left(-0.094\times 0.75Pa\right)}]$$

where Lg (°C) is the ground temperature, which is replaced with Ta (°C) in this model [38]. $R_{prim}$ refers to the solar radiation absorbed by the human body, which is calculated in the operating procedures designed by Bröde et al. [38].

### 2.4. Classification of Thermal Comfort Level

Based on the calculated UTCI temperature value, the comfort level of each city at any time can be divided according to the international classification standard (Table 1) [39]. In the annual thermal comfort analysis, three indicators were used: the annual UTCI average, the frequency of being at the thermal extreme comfort level (“hot/very hot/extremely hot”), and the frequency of being at the cold extreme comfort level (“cold/very cold/extremely cold”).

### 2.5. Landscape Pattern Index

The landscape pattern indices selected in this study included landscape percentage (PLAND), mean patch size (MPS), edge density (ED), area-weighted mean shape index (AWMSI), aggregation index (AI), maximum patch index (LPI), and Shannon diversity index (SHDI) (Table 2). Based on the remote-sensing monitoring data of land-use status in China (resolution 1 km × 1 km), an optimal pane size of 7 × 7 was selected in Fragstats 3.3 software to calculate the landscape pattern indices of five land-use types (1: cultivated land, 2: green land, 3: water area, 4: construction land, and 5: unused land) [40,41]. After standardized processing, Pearson correlation analysis was conducted between the thermal comfort index and the thermal comfort index using SPSS 25 software [42].

### 2.6. Geographic Detector

Geographic detectors are used to detect spatial heterogeneity and reveal the driving force behind them. The core idea is based on the assumption that if an independent variable (X) has an important influence on a dependent variable (UTCI), the spatial distribution of X and UTCI should be similar.

Compared to traditional regression analysis, the geographic detector abandons the linear relationship and identifies the degree of explanation of factors to dependent variables by differentiating spatial, that is, the differences between layers. Therefore, this method does not comply with the requirements of sample size and significance and avoids the problem of collinearity between factors [46,47,48,49]. Compared with the traditional linear regression method, this study used geographic detectors to identify the leading factors of annual and seasonal thermal comfort indices in Chinese cities.

In the calculation process, factor X must be discretized before it is used for driver identification in practice, and factor X should be stratified, classified, or partitioned. The discretized factor and dependent variable UTCI are then imported into the geographic detector for differentiation and factor detection. The main function is to detect the spatial differentiation of the dependent variable UTCI and the extent to which the detection factor X explains the spatial differentiation of attribute UTCI. The degree of explanation was measured using the statistical value *q*. The expression is as follows:

(4) $$q=1-\frac{\sum_{h=1}^{L}N_{h}\sigma_{h}^{2}}{N\sigma^{2}}$$

where h is the discretization result of factor X, corresponding to stratification, classification, or partition. $N_{h}$ is the number of variables in the h-layer, and N is the number of variables. $\sigma_{h}^{2}$ is the variance of the Y-value of the dependent variable in the *h*-layer, and $\sigma^{2}$ is the variance of the global dependent variable Y. The size of the q-value of the final output result is between 0 and 1. The larger the *q*-value, the greater the driving force of this factor on the spatial differentiation of the Y-value [50].

## 3. Results

### 3.1. Annual Thermal Comfort Evaluation of Cities in China

**Figure 3:**
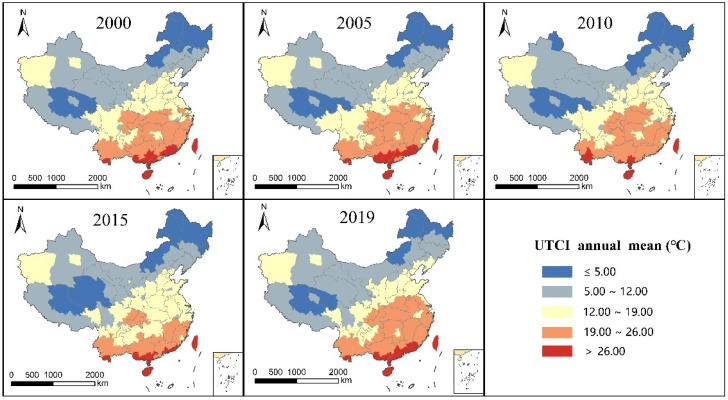
Annual mean distribution of UTCI in China from 2000 to 2020.

The average UTCI values of each city in the years 2000, 2005, 2010, 2015, and 2019 are statistically and visually displayed in Figure 3. The UTCI mean distribution pattern of the five years had the same general trend, showing a trend of high in the southeast and low in the northwest. The highest UTCI value was distributed in the southern part, whereas the lowest UTCI value was mainly distributed in the northeast and northwest of higher latitudes and in the southwest of Tibet at higher altitudes. In addition, the UTCI of the southern region of the Xinjiang Uygur Autonomous Region was significantly higher than that of the surrounding regions.

**Figure 4:**
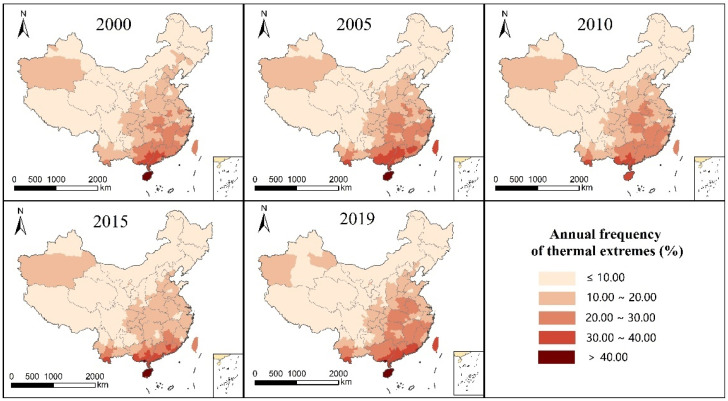
Annual frequency distribution of thermal extremes in China from 2000 to 2020.

The spatial distribution pattern of the frequency of annual thermal extreme thermal comfort in each city showed an obvious general trend of high in the southeast and low in the northwest in all five years (Figure 4). The highest frequency (more than 40.01%) was distributed in Hainan Province, which has the lowest latitude and highest annual temperature. The second-highest frequency values (between 30.01 and 40.00%) were mainly distributed in South China and southern Southwest China, where the latitude is also lower and the average annual temperature is higher. Cities with frequencies between 20.01 and 30.00 were mainly distributed in the south and the middle and lower reaches of the Yangtze River. These areas had a high frequency of extreme thermal comfort throughout the year, so attention should be paid to improving the living environment.

**Figure 5:**
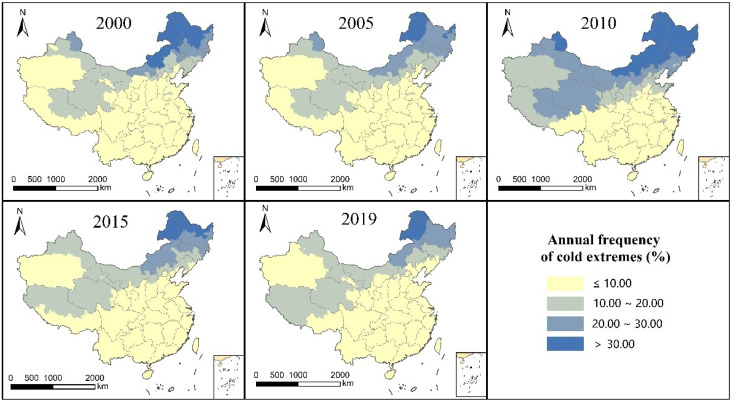
Annual frequency distribution of cold extremes in China from 2000 to 2020.

As can be seen from Figure 5, the spatial distribution pattern of frequency of cold extreme comfort grade in different years was basically the same, showing a trend of low in the southeast and high in the northwest. Most regions of South China, Central China, East China, and Southwest China showed low values, mainly due to relatively high winter temperatures in these regions and a lower frequency of cold extremes. The southern part of the Xinjiang Uygur Autonomous Region in Northwest China also showed low values, mainly because it is located in a basin and the high mountains in the north block the invasion of cold air from the north and reduce the occurrence of extreme cold weather. The high-frequency values mainly appeared in the northern region with high latitudes and the Qinghai-Tibet Plateau at high altitudes.

The three annual analysis indicators (average annual UTCI, extreme comfort level in thermal frequency, and frequency of extreme cold comfort rank) of the spatial distribution pattern presented the following pattern: (1) For the same index, the spatial distribution of each year trend is consistent, and the spatial distribution pattern of change is not large and is not affected by the year of change. (2) The annual UTCI average and annual frequency in the extreme thermal comfort level show an overall trend of high in the southeast and low in the northwest, and the annual frequency in the cold extreme comfort level shows an overall trend of low in the southeast and high in the northwest. The distribution trend is consistent with the characteristics of climate distribution in China. More attention should be paid to the southern region with a high frequency of extreme thermal comfort and the northern region with a high frequency of cold extreme comfort, particularly in the northeast region. (3) The low-value distribution area of UTCI and the high-value distribution area of cold extreme thermal comfort level frequency coincide, and the high-value distribution area of UTCI and the high-value area of thermal extreme thermal comfort level are also consistent. This indicates that there is no significant difference in the spatial distribution pattern between the UTCI mean and frequency for extreme comfort from the perspective of annual indicators. (4) The UTCI mean value, thermal extreme comfort frequency, and cold extreme comfort frequency of the southeastern Xinjiang Uygur Autonomous Region are higher than the surrounding values.

### 3.2. Spatial and Temporal Distribution of Urban Thermal Comfort during Heat Wave in China

#### 3.2.1. Identification of Heat Wave Period

The criteria for identifying heat wave periods in this study were as follows: The maximum temperature exceeded 25 °C for at least five consecutive days, and the maximum temperature exceeded 30 °C for at least three days [51]. Through the statistical analysis of the temperature data of Chinese cities from June to September 2019, it was found that the number of cities in the heat wave period was the largest for five consecutive days from 24 July 2019 to 28 July 2019. During this period, 70% of the cities met the above criteria for the heat wave period; therefore, this study selected 24–28 July 2019 as a typical heat wave period.

**Figure 6:**
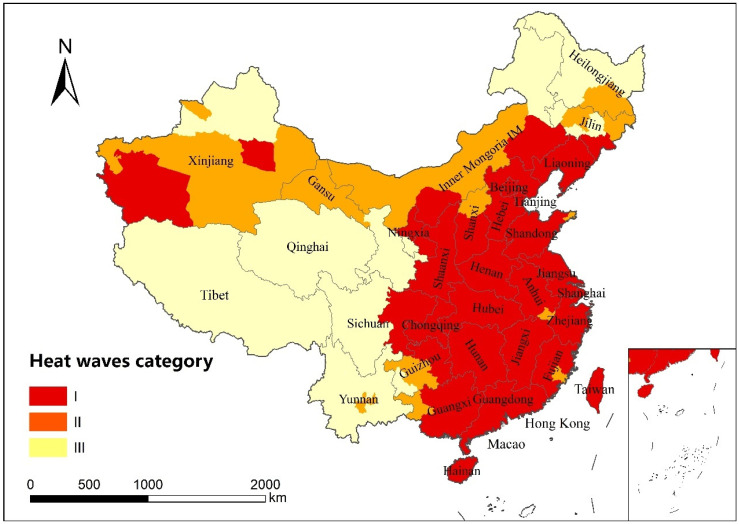
Distribution of cities during the heat wave period from 24 July 2019 to 28 July 2019.

In the selected typical heat wave period, cities were classified according to temperature data into the following three categories: Class I cities were in the heat wave period, with five days exceeding 25 °C and at least three days exceeding 30 °C. The temperature of Class II cities was relatively high, but did not meet the standard of a heat wave period. All five days exceeded 25 °C, but fewer than three days exceeded 30 °C. The temperature of Class III cities was relatively low and did not exceed 25 °C for all five days. According to the classification results, 240 cities fully met the criteria of being in a heat wave period, mainly distributed in East China and the Xinjiang Uygur Autonomous Region in Northwest China. There were 31 cities with high temperatures that did not meet the criteria for heat waves, and the distribution was scattered, both in the south and north. There were 73 cities with low temperatures, mainly in the northern and southwestern regions (Figure 6).

#### 3.2.2. Spatial Distribution of Thermal Comfort during Heat Wave

**Figure 7:**
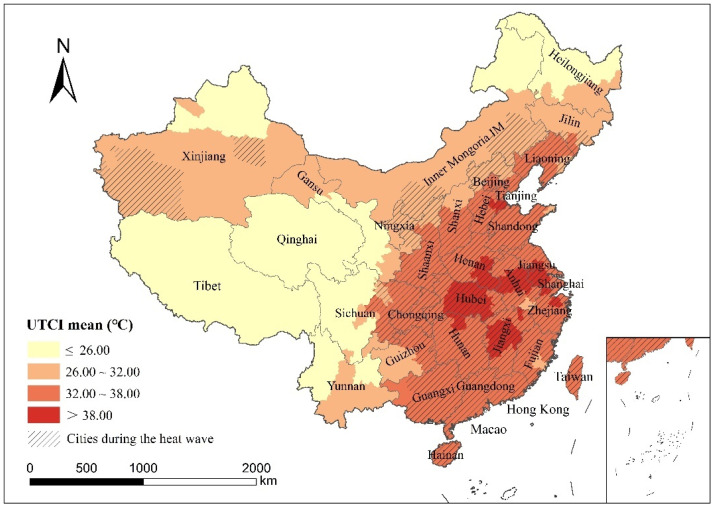
Mean UTCI distribution during the heat wave (shaded areas are cities during the heat wave).

The UTCI values and corresponding thermal comfort levels of the five selected moments (8:00, 11:00, 14:00, 17:00, and 20:00) with frequent human activity during the typical heat wave were analyzed. According to the UTCI classification standards for thermal comfort, the UTCI average values of each city during the heat wave were divided into four levels (Figure 7).

The UTCI average during the heat wave showed an obvious trend of higher in the southeast and lower in the northwest. There were 39 cities with a UTCI average >38 °C (corresponding to “very hot” thermal comfort level), distributed in the middle and lower reaches of the Yangtze River and the Beijing–Tianjin–Hebei region. This is because the sub-high-pressure belt moves to the Yangtze River area in summer, the airflow sinks, and the temperature rises in the middle and lower reaches of the Yangtze River. However, the Beijing–Tianjin–Hebei region has long sunshine duration, high energy demand and consumption, high urban surface temperature, and a significant urban heat island effect. There were 184 cities with UTCI between 32.01 and 38.00, which were distributed in East China. This is because the eastern region is located in a subtropical monsoon climate and temperate monsoon climate zone with high temperatures and rainy summers. There were 52 cities with UTCI <26 °C, distributed in Northeast, Northwest, and Southwest China. Because of the high latitude or altitude, the temperature of these cities was low, and the maximum temperature was not greater than 25 °C during the heat wave, so the comfort was strong.

Superposition analysis of UTCI mean distribution and cities identified as being in heat wave period showed that cities identified as being in a heat wave period had higher UTCI mean values, and the UTCI mean values were generally above 32 °C (corresponding to the thermal comfort level of “hot” and “very hot”), indicating that the high distribution of UTCI values showed strong consistency with air temperature. However, the average value of cities identified as being in a heat wave period in the Xinjiang Uygur Autonomous Region, Inner Mongolia, and other regions was low, which may be because the recognition of the heat wave period only focused on the maximum temperature, whereas the UTCI average value was calculated multiple times.

**Figure 8:**
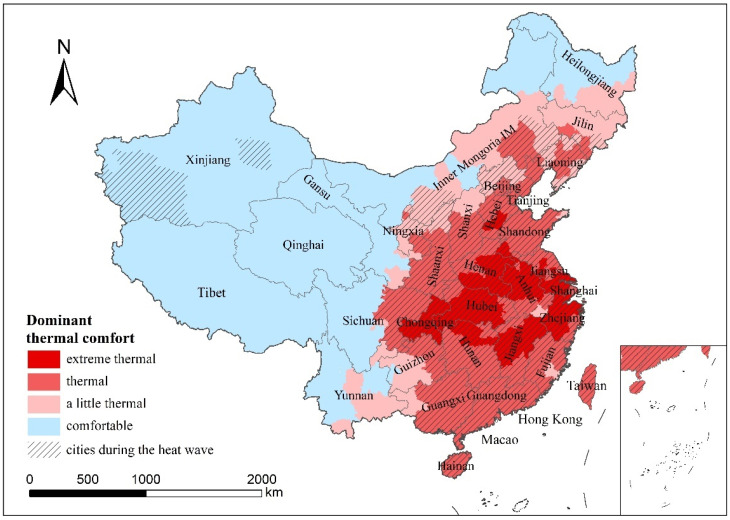
Distribution of dominant thermal comfort levels in different cities during the heat wave (shaded areas are cities during the heat wave).

To avoid the influence of intraday or diurnal differences in UTCI values, the thermal comfort level with the highest frequency during the heat wave in each city was taken as the dominant thermal comfort level of the corresponding city, as shown in Figure 8. The regions with “very hot” thermal comfort were mainly located in the Beijing–Tianjin–Hebei region and the eastern part of Central China. Similar to the distribution of the UTCI mean value, the regions with a dominant thermal comfort grade of “hot” were also concentrated in East China. However, the distribution area with the dominant thermal comfort rating of “relatively hot” was smaller and more concentrated than the UTCI average. The dominant thermal comfort in a large area of Western China was “comfortable,” whereas the dominant thermal comfort in Northeast China was consistent with the UTCI average.

**Figure 9:**
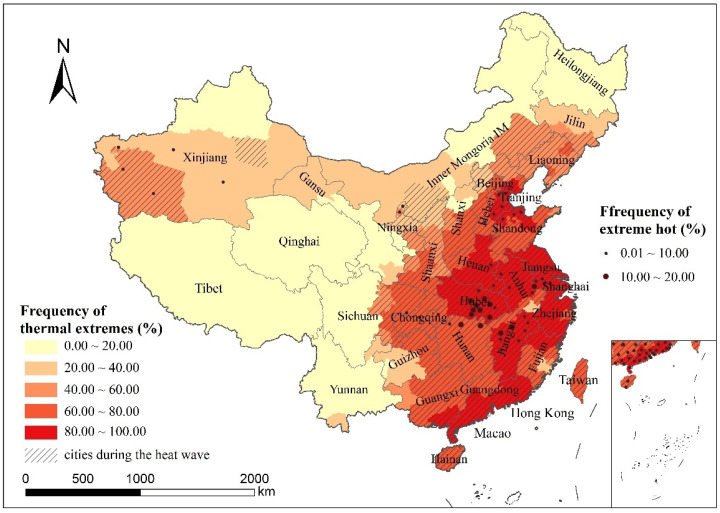
Frequency distribution of thermal extreme comfort levels during heat waves (shaded areas are cities during the heat wave).

Figure 9 shows the frequency spatial distribution of “extremely hot” and “hot/very hot/extremely hot” extreme thermal comfort levels in each city during heat waves. For the frequency of “hot/very hot/extremely hot,” the distribution of cities in China still showed a general trend of high in the southeast and low in the northwest, which is consistent with the distribution of UTCI mean and dominant comfort level. The Beijing–Tianjin–Hebei region and the middle and lower reaches of the Yangtze River were still highly distributed areas due to the influence of high temperatures caused by the subtropical high-pressure belt. There was also a large area of high value in Guangdong province, mainly because the region belongs to the subtropical monsoon climate zone, where human economic activity is concentrated. Cities with “extremely hot” thermal comfort were mostly located in the Beijing–Tianjin–Hebei region and the middle and lower reaches of the Yangtze River. Xinjiang and northern Ningxia also showed “extremely hot” grade—the southern region of Xinjiang in particular had a high frequency of “hot/very hot/extremely hot”—but the average UTCI value of these regions was at a low level, indicating that the difference in UTCI value between day and night might be large in these regions, so we should pay more attention to the outdoor thermal comfort during daytime high temperature.

### 3.3. Attribution of Spatial Heterogeneity of Thermal Comfort

Based on the optimal discretization results given in Appendix A, each factor that could pass the significance test at the 0.01 level and the corresponding optimal category number were selected for this study, and the heterogeneous attribution analysis of each dependent variable was completed using geographic detectors.

**Figure 10:**
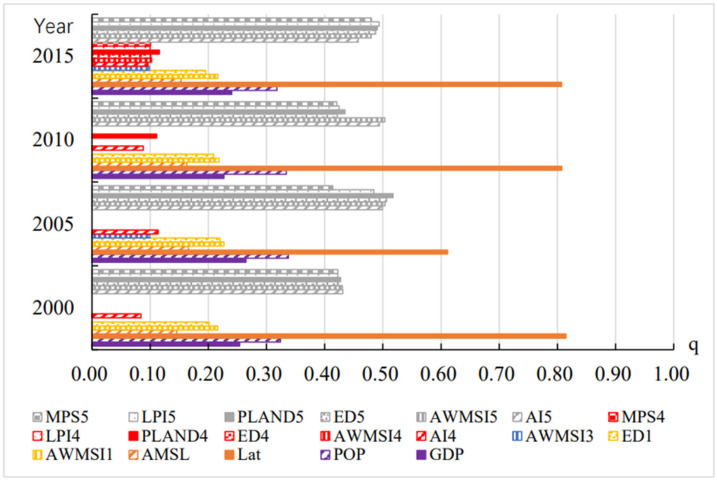
Q statistics between UTCI annual mean and driving factors (1: cultivated land; 2: green space; 3: water bodies; 4: construction land; 5: unused land).

The Q statistics of annual UTCI mean drivers were obtained using geographic detectors (Figure 10). It was found that latitude (Lat) was the dominant factor in the annual UTCI mean for 2000, 2005, 2010, and 2015, and accounted for 60% and 80% of the annual UTCI mean. Second, the Q values of the six landscape pattern indices of the unused land could explain the UTCI average by more than 40%. Finally, the socioeconomic factors GDP and POP, natural factor elevation (AMSL), and landscape pattern indices AWMSI and ED of cultivated land type were explained with a power between 20% and 40%. The landscape pattern index of construction land type, especially the AI index, also showed explanatory power for each year.

**Figure 11:**
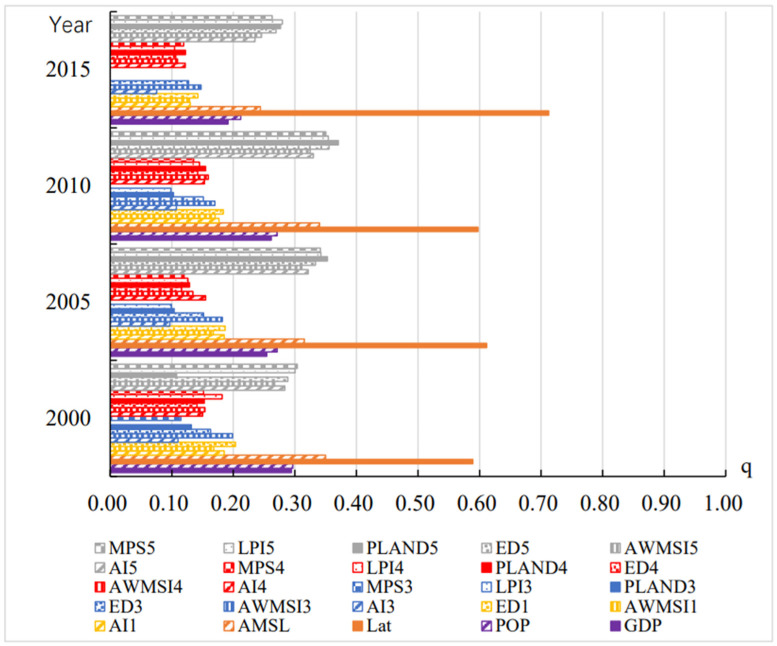
Q statistics between annual frequency of extreme thermal comfort and driving factors (1: cultivated land; 2: green space; 3: water bodies; 4. construction land; 5: unused land).

Figure 11 shows the driving factors of the heterogeneity in the frequency of extreme thermal comfort in Chinese cities. It can be seen from the figure that the leading factors of the frequency of thermal extreme comfort are all natural factors of latitude, and the latitude factor has a significantly higher explanatory degree than other factors. The q-values of altitude, socioeconomic factors, GDP and POP, and the landscape pattern index of unused land are next, ranging from 20% to 40%. In addition, the landscape pattern indices of cultivated land, water areas, and construction land also have 10–20% explanatory power, which are also important driving factors of frequency heterogeneity of annual thermal extreme comfort.

**Figure 12:**
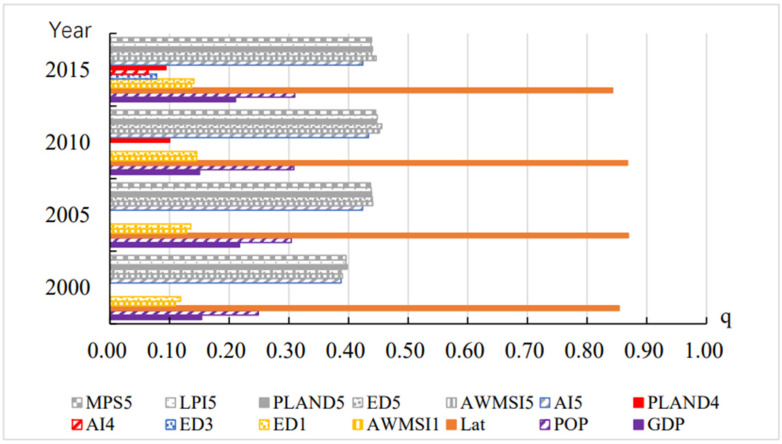
Q statistics between annual frequency of extreme cold comfort and driving factors (1: cultivated land; 2: green space; 3: water bodies; 4: construction land; 5: unused land).

The heterogeneous attribution results for the annual cold extreme comfort frequencies are plotted in Figure 12. Latitude was still the dominant factor in the frequency of cold extreme comfort, and the explanation of annual cold extreme frequency was up to 80%, which is much higher than other factors. The q-value of the landscape pattern index of unused land was second only to the latitude factor, with an explanatory power of 35%. The explanatory power of the population factor POP was greater than 20%, and the explanatory power of the economic factor GDP was between 10% and 20%. The explanatory power of the landscape pattern index of cultivated land was greater than 10%. However, the natural altitude factor failed to pass the significance test of 0.01.

In conclusion, for each annual index, the relative explanatory power of different driving factors on the annual index is relatively consistent in different years, especially for driving factors with strong explanatory power. Both the UTCI mean and extreme comfort frequency are driven by latitude, indicating that latitude plays a leading role in the spatial differentiation pattern of the annual urban thermal comfort index in China. In addition, GDP and POP are important driving factors that can explain the spatial variation in each annual indicator to a certain extent. Among the landscape pattern indices, those of unused land have a greater explanatory power for each annual index. In addition, altitude does not pass the significance test as an important driver of the frequency of cold extreme comfort, although it shows high explanatory power for the annual UTCI mean and extreme thermal comfort frequency.

## 4. Discussion

The following three problems exist in studies of outdoor thermal comfort. First, the spatial scale of thermal comfort research is small, and the research scope is usually community, park, or a single city, etc., which lacks large-scale research, especially thermal comfort research in China [52,53,54,55,56,57], resulting in a lack of comparative analysis of thermal comfort evaluation results in different regions under different climate change backgrounds. Second, most studies only evaluate thermal comfort in a single period (most studies select the heat wave period), lacking long-term series and monitoring in different seasons [53,54,58]. Third, although some studies use long-term meteorological data, these studies usually use daily or monthly average data [14]. This may lead to a situation where extreme temperatures are erased and extreme climate phenomena are ignored, and it is difficult to comprehensively and meticulously evaluate the thermal comfort of the study area. Therefore, this study expands the space and time dimensions to obtain more comprehensive and systematic evaluation results.

During the study period, the spatial pattern and differentiation characteristics of the annual analysis indices in different years were similar, and the inter-annual differences were small. During a typical heat wave period (from 24 July 2019 to 28 July 2019), 70% of the cities were in the heat wave period, mainly distributed in East and Northwest China’s Xinjiang Uygur Autonomous Region. The UTCI mean high value and dominant comfort level were “very hot,” which were mainly distributed in the middle and lower reaches of the Yangtze River and the Beijing–Tianjin–Hebei region, which is also reflected in previous studies [59,60]. A possible reason for the poor thermal comfort in the middle and lower reaches of the Yangtze River during heat waves is that the secondary high-pressure belt moves to the Yangtze River area in summer and the air flow sinks, leading to a temperature rise, decreased rainfall, dry climate, and increased solar radiation [61,62]. In the Beijing–Tianjin–Hebei region, owing to the high latitude, long sunshine duration, sufficient solar radiation, low and flat terrain, and poor air mobility, the thermal comfort of the region is poor in extreme thermal climates [63,64].

At the national level, the driving factors affecting the spatial distribution of heat and cold comfort differed to some extent. The natural factors of latitude and altitude were the important factors affecting the spatial differentiation of the urban comfort level [65,66,67,68]. For regions with high latitudes and low altitudes, more attention should be paid to the thermal comfort [68]. In addition, in the process of urban development, attention should be paid to the possible impact of GDP and POP growth on urban thermal comfort, and reasonable planning and layout measures should be implemented to improve urban thermal comfort [69,70,71]. For areas to improve thermal comfort in summer, such as the middle and lower reaches of the Yangtze River, the Beijing–Tianjin–Hebei region, and southern Guangdong, attention should be paid to the layout of construction land, water bodies, and green space types and the overall layout at the landscape level [43,72], and corresponding measures should be formulated to achieve the goal of improving the thermal environment.

It is necessary to pay attention to outdoor thermal comfort under extreme climate conditions, improve the climate comfort of outdoor activity areas, and provide a good living environment [14,39]. Taking corresponding summer measures in outdoor areas to protect the health of residents in outdoor activities and operations can reduce people’s loneliness [73] and have a significant positive impact on the lifespan of the urban elderly [74,75], which can also reduce the cooling load of buildings and the utilization rate of electronic equipment such as air conditioning [76,77]. For regions showing extreme thermal comfort during heat waves, including the middle and lower reaches of the Yangtze River, Beijing–Tianjin–Hebei region, southern Guangdong, Xinjiang Uygur Autonomous Region, and Ningxia Autonomous Region, the prediction and early warning of thermal extremes should be strengthened. For the southeastern regions with poor thermal comfort in summer, and the northeastern and northwestern regions with cold thermal comfort in winter, some measures should be taken to prevent heat and cold. For example, cool/green roofs and urban greening can reduce the moderate urban thermal environment and urban heat island phenomena [15], and optimized design of urban spaces and appropriate microclimatic planning can increase the thermal comfort [78].

The results of this study can provide a reference for establishing effective measures to improve urban outdoor thermal comfort, but there are still two deficiencies in this study: (1) The longitudinal span of China is large, so a more reasonable comparison and analysis should be made considering the actual time difference between the eastern and western regions. However, owing to data limitations, this study can only obtain the historical data of meteorological stations with an interval of 3 h, so the effect of time difference cannot be excluded in the actual study. (2) In heterogeneity attribution analysis, more potential drivers should be included to improve the accuracy of the results, such as human subjective thermal state factors and the number of urban motor vehicles. In subsequent studies, more advanced data acquisition methods will be adopted for more accurate analysis.

## 5. Conclusions

Based on the UTCI index, this study evaluated the thermal comfort of 344 municipal administrative regions in China at 8:00, 11:00, 14:00, 17:00, and 20:00, with high human activity intensity, in 2000, 2005, 2010, 2015, and 2019, respectively. The spatial pattern of thermal comfort in Chinese cities was studied in detail using relevant indicators during heat waves and annual analysis indicators, and the driving factors of spatial differentiation of thermal comfort in Chinese cities were identified based on this. The main conclusions are as follows:

(1) The spatial patterns and differentiation characteristics of the three types of annual analysis indicators were similar in different years, and the inter-annual differences were small. The annual UTCI values showed a general trend of higher in the southeast and lower in the northwest. The annual frequency of extreme thermal comfort also showed a trend of high in the southeast and low in the northwest. The annual frequency of cold extremes showed a trend of low in the southeast and high in the northwest.
(2) The analysis of the typical heat-wave period showed that the cities in the heat-wave period were mainly distributed in the Xinjiang region in East and Northwest China. The UTCI mean value and frequency of extreme thermal comfort (“hot/very hot/extremely hot”) showed a general trend of high in the southeast and low in the northwest during the heat wave. The dominant comfort level also showed a trend of hot in the southeast and comfortable in the northwest. In high-temperature-warning weather, the corresponding local government departments should take relevant measures to prevent heat stroke, reduce temperature, and ensure the thermal comfort of outdoor activity spaces.
(3) In different years, the relative contribution rates of the driving factors of the same index were basically the same and were not affected by year changes, especially for factors with high contribution rates. The leading factor for the annual indicators was latitude. In addition, the socioeconomic factors GDP and POP, as well as the landscape pattern index of unused land type and cultivated land type, all showed a high explanatory degree for each annual indicator.

## Figures and Tables

**Table 1:** Classification standard of thermal comfort based on UTCI.

UTCI	Thermal Stress Category	Comfort Level	UTCI	Thermal Stress Category	Comfort Level
>46	Extreme heat stress	Extremely hot	0–9	Slight cold stress	Cool
38–46	Strong heat stress	Very hot	−13–0	Moderate cold stress	Slight cold
32–38	Strong heat stress	Hot	−27–13	Strong cold stress	Cold
26–32	Moderate heat stress	Slight heat	−40–27	Very strong cold stress	Very cold
9–26	No thermal stress	Comfortable	<−40	Extreme cold stress	Extremely cold

**Table 2:** Description of the selected landscape pattern indices [43,44,45].

Index	Explanation	Value Range
Landscape percentage (%)	$PLAND=p_{i}=\frac{\sum_{j=1}^{n}a_{ij}}{A}\times 100$$,\;a_{ij}$ is the area of patch *ij*, *A* is the total area of landscape. Represents the abundance ratio of a patch type in the landscape.	[0,100]
Mean patch size (ha)	$MPS=\frac{\sum_{j=1}^{n}a_{ij}}{n}$$,a_{ij}$ is the area of plaques ij, and *n* is the number of plaques of this type. Represents the average area of a patch type.	>0
Edge density (m/ha)	$ED=\frac{\sum_{i=1}^{M}\sum_{j=1}^{M}P_{ij}}{A}$$,P_{ij}$ is the boundary length between the patches of type *i* and the adjacent patches of type *j*, and *A* is the total landscape area. Represents the edge length between patches of different landscape elements in a unit area.	>0
Area-weighted mean shape index	At plaque level, it is the sum of the peripheral-area ratio of each patch in a patch type multiplied by their respective area weight.	≥1
Aggregation index (%)	$AI= [\frac{g_{ii}}{maxg_{ii}}]\times 100$$,g_{ii}$$\;\mathrm{is}\;\mathrm{the}\;\mathrm{number}\;\mathrm{of}\;\mathrm{nodes}\;\mathrm{before}\;\mathrm{patch}\;\mathrm{type}\;i\;\mathrm{pixel},\;maxg_{ii}$ is the maximum number of nodes before patch type *i* pixel. Represents the degree of aggregation between plaques.	[0,100]
Largest patch index (%)	$LPI=\frac{maxa_{i}}{A}$$,maxa_{i}$ is the area of the largest patch in a patch type, *A* is the total landscape area. Represents the proportion of the largest patch in a patch type to the landscape area.	(0,100]
Shannon diversity index	Represents landscape heterogeneity. The richer the land use types, the higher the value.	≥0

## Appendix A

According to the working principle of the geographic detector, before using it for attribution analysis, the values of each independent variable must be discretized and graded, and then related factor detection and spatial statistical analysis can be carried out. In this study, several experiments were conducted on commonly used discretization methods, including the K-means clustering, natural breakpoint, and bisection methods. Finally, it was determined that the discretization method with the highest q-value had the best effect. Therefore, this study paired each independent variable and each dependent variable that had a significant correlation with it, conducted experiments on the number of categories selected by the independent variables in each pair of combinations in the process of discretization, and obtained the optimal number of categories for each combination. The results are presented in Table A1, Table A2 and Table A3.

**Table A1:** The optimal number of categories for discretization of the influence factors of the annual UTCI mean for each year (** represents *p* < 0.01, * represents *p* < 0.05; 1: cultivated land; 2: green space; 3: water bodies; 4. construction land; 5: unused land).

	2000	2005	2010	2015
GDP	15 **	12 **	15 **	11 **
POP	15 **	15 **	15 **	15 **
Lat	15 **	3 **	15 **	15 **
AMSL	15 **	15 **	15 **	15 **
AI(4)	13 **	14 **	14 **	11 **
AI(5)	3 **	5 **	5 **	5 **
AWMSI(1)	15 **	15 **	15 **	15 **
AWMSI(3)	15 *	14 **		15 **
AWMSI(4)				15 **
AWMSI(5)	3 **	10 **	10 **	11 **
ED(1)	15 **	15 **	15 **	13 **
ED(4)		14 *	15 *	13 **
ED(5)	3 **	8 **	3 **	11 **
PLAND(4)		15 *	15 **	15 **
PLAND(5)	3 **	10 **	3 **	12 **
LPI(4)		14 *	15 *	14 **
LPI(5)	3 **	6 **	3 **	12 **
MPS(4)		14 *	15	15 **

**Table A2:** The optimal number of categories for discretization of the influence factors of the annual thermal extreme comfort frequency in each year (** represents *p* < 0.01, * represents *p* < 0.05; 1: cultivated land; 2: green space; 3: water bodies; 4. construction land; 5: unused land).

	2000	2005	2010	2015
GDP	15 **	13 **	15 **	14 **
POP	14 **	11 **	15 **	10 **
Lat	15 **	15 **	15 **	15 **
AWSL	14 **	14 **	14 **	13 **
AI(1)	15 **	15 **	15 **	15 **
AI(3)	15 **	14 **	13 **	9 **
AI(4)	13 **	14 **	14 **	11 **
AI(5)	5 **	5 **	5 **	5 **
AWMSI(1)	15 **	15 **	15 **	15 **
AWMSI(4)	15 **	15 **	15 **	15 **
AWMSI(5)	9 **	10 **	10 **	11 **
ED(1)	14 **	15 **	15 **	15 **
ED(3)	15 **	13 **	13 **	11 **
ED(4)	15 **	14 **	15 **	13 **
ED(5)	10 **	10 **	10 **	11 **
PLAND(3)	14 **	12 **	12 **	14 *
PLAND(4)	15 **	15 **	15 **	15 **
PLAND(5)	15 **	10 **	10 **	12 **
LPI(3)		14 **	15 **	
LPI(4)	15 **	15 **	15 **	14 **
LPI(5)	10 **	10 **	10 **	12 **
MPS(3)	15 **			
MPS(4)	15 **	15 **	15 **	15 **
MPS(5)	10 **	10 **	10 **	10 **

**Table A3:** The optimal number of categories for discretization of the influence factors of the annual cold extreme comfort frequency in each year (** represents *p* < 0.01, * represents *p* < 0.05; 1: cultivated land; 2: green space; 3: water bodies; 4: construction land; 5: unused land).

	2000	2005	2010	2015
GDP	15 **	12 **	15 **	15 **
POP	10 **	11 **	15 **	11 **
Lat	15 **	15 **	15 **	15 **
AMSL	13 *	13 *	13 *	13 *
AI(4)		14 *	14	9 **
AI(5)	5 **	5 **	5 **	4 **
AWMSI(1)	13 **	15 **	13 **	15 **
AWMSI(3)	10 *	15	15 *	15 *
AWMSI(4)				15
AWMSI(5)	10 **	10 **	10 **	11 **
ED(1)	12 **	15 **	12 **	14 **
ED(3)	15	13 *	13 *	12 **
ED(4)		14	15	14
ED(5)	8 **	8 **	8 **	8 **
PLAND(4)		15	15 **	14 **
PLAND(5)	10 **	8 **	10 **	12 **
LPI(4)		15	12 *	14
LPI(5)	8 **	10 **	10 **	10 **
MPS(4)				12 *
MPS(5)	10 **	10 **	10 **	11 **
